# Balancing Innovation and Patient Care in Breast Cancer: Integrating Hypofractionated Proton Therapy With Breast Reconstruction Outcomes

**DOI:** 10.7759/cureus.58056

**Published:** 2024-04-11

**Authors:** Hadia Fatima, Paras Abbas, Salem M Alshehri

**Affiliations:** 1 Radiation Oncology Department, King Abdulaziz Medical City, Ministry of National Guard Health Affairs, Riyadh, SAU; 2 Oncology Department, Atomic Energy Cancer Hospital, Nuclear Medicine Oncology and Radiotherapy Institute, Islamabad, PAK

**Keywords:** proton beam radiotherapy, multidisciplinary cancer care, breast implants, reconstructive breast surgery, breast cancer research

## Abstract

This review aims to assess the application of hypofractionated proton therapy in breast cancer reconstruction, analyzing its advantages, challenges, and broader implications for patient care. The goal is to comprehensively understand how this innovative approach can be integrated into breast cancer treatment. Proton therapy exhibits superior target coverage and safety, reducing radiation-induced complications and sparing critical organs, but skin toxicity outcomes differ from photon therapy. Tissue expanders are vital in breast reconstruction, employing innovative planning for positive long-term outcomes and highlighting the importance of balancing cancer treatment effectiveness with cosmetic outcomes. Hypofractionated proton therapy and breast cancer reconstruction present promising innovations with notable advantages in target coverage and organ sparing. However, variations in skin toxicity outcomes and the need for a careful balance between treatment effectiveness and cosmetic outcomes underscore ongoing challenges. Future directions should focus on refining treatment protocols, optimizing patient selection criteria, and integrating emerging technologies to enhance therapeutic outcomes while minimizing adverse effects.

## Introduction and background

Breast cancer ranks as the most prevalent malignant tumor among women globally, comprising 36% of oncological cases [1], with 2.26 million new cases reported in 2020 [2]. According to the World Health Organization (WHO), malignant neoplasms, particularly breast cancer, impose a significant burden on women, contributing to an estimated 107.8 million disability-adjusted life years [2].

The role of radiotherapy in breast cancer management has been highlighted by studies revealing a threefold reduction in local recurrence rates when combined with surgery. However, the addition of radiotherapy may not significantly alter the 10-year survival rates [3]. Over the past two decades, extensive research has explored radiotherapy's role in cancer treatment, with approximately 60-70% of cancer patients undergoing photon therapy delivered by linear accelerators [4]. Proton therapy, a form of ion-beam particle therapy, has gained prominence due to its physical advantages, including the Bragg peak. With over 70 proton therapy centers globally and more than 190,000 patients undergoing proton therapy, these facilities contribute significantly to large cooperative clinical trials and the expanding scientific literature on proton therapy, marking a promising frontier in cancer treatment [4].

Factors associated with postmastectomy reconstruction timing, such as preoperative factors (procedure type and number of interventions), have no impact on complications or quality of life. Immediate and delayed reconstructions have shown comparable quality-of-life outcomes in patients undergoing mastectomy for breast cancer [5]. This review aims to assess the application of hypofractionated proton therapy in breast cancer reconstruction, analyzing its advantages, challenges, and broader implications for patient care. The goal is to comprehensively understand how this innovative approach can be integrated into breast cancer treatment.

## Review

Methods

A comprehensive literature review spanning from 1995 to 2023 was done, utilizing PubMed, Google Scholar, and Scopus. Key terms such as "breast cancer," "proton therapy," "breast reconstruction," and "hypofractionation" were employed to identify relevant studies and ongoing clinical trials from sources like ClinicalTrials.gov. The analysis focused on dosimetric advantages, safety profiles, cardiac sparing, dose distribution, critical structures, skin toxicity, and patient-centered care (PCC). The multidisciplinary nature of breast cancer management was considered, incorporating insights from oncoplastic surgery, intraoperative radiation therapy, and neoadjuvant radiation therapy.

Proton therapy fundamentals

Particle beam irradiation, specifically proton therapy, is a type of external beam radiotherapy with several benefits over traditional photon radiotherapy. It is beneficial for cancer patients in two common scenarios. Firstly, because there is no exit dose, it can deliver therapeutic radiation precisely to tumors located in difficult anatomical positions. Secondly, it can reduce the integral dose (low-dose bath) to normal tissues surrounding the tumor, which may lower the risk of late toxicities and secondary cancers [6]. Protons, due to their highly conformal delivery, allow for greater sparing of normal tissues and the potential escalation of tumor doses, which can result in improved treatment outcomes. Recent and ongoing research has uncovered additional advantages of proton therapy. Notably, the higher relative biological effectiveness (RBE) near the end of the proton range can be used to enhance the disparity in biologically effective doses (BEDs) between tumors and normal tissues. Additionally, the compact nature of proton dose distributions, referred to as the "dose bath," has reduced exposure to circulating lymphocytes and immune organs at risk (OARs) [7].

In proton therapy, therapeutic protons are accelerated to energies ranging from 70 to 250 MeV and transported to the treatment room, where they enter a rotating gantry-mounted treatment head. The initially narrow proton beams undergo lateral and longitudinal spreading and shaping to deliver treatments effectively. This can be achieved through electro-mechanical methods for "passively scattered proton therapy" or through magnetic scanning of thin "beamlets" in the case of optimized intensity modulated proton therapy, which is becoming the predominant proton therapy modality [8].

Proton therapy's effectiveness is limited due to anatomical variations, approximations for dose computation, and assumptions about RBE. While it's effective for certain cancers, there is insufficient evidence to support its broader application. Protons exhibit an inverted dose profile, reducing the radiation dose to normal tissues and resulting in fewer side effects [9].

Clinical applications of proton therapy in breast cancer

Proton therapy is increasingly favored for breast cancer treatment, offering reduced radiation exposure to surrounding tissues. Despite established dosimetric benefits, the long-term clinical outcomes necessitate further research. Conducting randomized trials comparing proton and photon therapy faces challenges due to logistical, financial, and ethical considerations.

Mutter et al. analyzed 13 articles, comprising six on passive proton therapy (double scattering), five on pencil beam scanning (PBS), and two combining both techniques. Proton therapy consistently demonstrated superior target coverage compared to photons, even against intensity-modulated radiation therapy (IMRT). Proton therapy minimized volumes receiving 105% of the dose, achieving volumes receiving 95% of the dose at approximately 98%. Remarkably, proton therapy consistently reduced mean heart dose (MHD) by a factor of 2 or 3, i.e., 1 Gy with proton therapy versus 3 Gy with conventional 3D, and 6 Gy for IMRT. Lungs were also better preserved with proton therapy. Cutaneous toxicity observed with double scattering improved with proton therapy [10]. Proton therapy planning requires factoring range uncertainty, typically expressed as a percentage of the proton's incident range. Breast treatment has shallow treatment ranges and lower uncertainties in range calculations because the tissues traversed are mostly fatty, muscular, or glandular. To create robust proton therapy plans that account for range uncertainty, composite distal margins of approximately 2 to 3 mm are often applied. This approach allows for precise sparing of the underlying heart and lungs [10]. Breast cancer treatment with proton beam therapy (PBT) offers distinct advantages (Table 1).

**Table 1:** Potential distinct advantages of PBT in breast cancer PBT: proton beam therapy [11,12]

Anatomical considerations	High-risk scenarios	Reconstruction and contouring
Large target volumes	Patients at high risk for contralateral breast cancer with underlying genetic mutations	Sparing of the vascular anastomosis in patients who underwent breast reconstruction with tissue flaps
Inflammatory breast cancer	Rare mutations carry enhanced sensitivity to radiation therapy	Lower the risk for lymphedema by enhancing the sparing of dissected axilla in appropriate situations
Deep nodal boost	Germline TP53 mutations predispose to primary malignancy, treatment-related malignancy, and up to a 33% risk of radiation-induced sarcoma in women undergoing breast radiotherapy	Modest data on decreasing the chances of reconstructive loss attributable to radiotherapy
Extensive skin involvement	In the setting of re-irradiation	Internal mammary nodal coverage lying immediately adjacent to the heart
Bilateral breast cancer	Patients at high risk for developing late radiation-related toxicities	
Advanced-age patients with underlying cardiac risk factors or the presence of hypertension, diabetes, hypercholesterolemia, and smoking		
Pectus excavatum		
Oligometastatic breast cancer with contiguous sternal or mediastinal oligomets		
Adequate coverage of the medial portion of the supraclavicular fossa		

Hypofractionated proton therapy

Hypofractionated proton therapy, a newer approach, uses larger, less frequent doses, shortens treatment time, and delivers high doses of proton beams to breast tumors in fewer treatment sessions compared to conventional radiation therapy. The rationale behind hypofractionated proton therapy in breast cancer lies in its ability to escalate the biologically effective dose (BED) to tumor cells while reducing treatment duration. By delivering higher doses of radiation per session, hypofractionation enhances tumor control rates while minimizing the risk of long-term side effects. Additionally, shorter treatment courses improve patient convenience and compliance, leading to better overall outcomes. The phenomenon of the Bragg peak enables clinicians to tailor radiation doses to the size, shape, and depth of breast tumors, optimizing treatment efficacy while sparing critical organs. Hypofractionated proton therapy has demonstrated efficacy in various breast cancer scenarios, including post-mastectomy radiation, partial breast irradiation, and reirradiation for recurrent disease [12].

Hypofractionated proton therapy, exemplified by the MC 1631 trial, condenses treatment, enhancing convenience, patient compliance, and resource efficiency [13]. However, careful patient selection is crucial, especially with immediate breast reconstruction (IBR), considering potential complications. A thorough evaluation of each patient's case and risk factors determines optimal reconstruction timing (Table 2).

**Table 2:** Prospective trials addressing hypofractionated proton therapy's impact on breast cancer reconstruction outcomes PMRT: post-mastectomy radiotherapy, RT: radiotherapy, IBR: immediate breast reconstruction

Name of the study	Type of study	Scope of the study	Fractionation protocols used	Results
MC-1631 [13]	Phase II	Comparison of conventional fractionation and hypofractionation in patients with indications for PMRT, including those with IBR.	Patients were randomly assigned to either conventional fractionation (50 Gy in 25 fractions) or hypofractionation (40.05 Gy in 15 fractions).	After a median follow-up of 39.3 months, the study could not establish non-inferiority of hypofractionation. However, it was noted that hypofractionated proton PMRT appeared to be tolerable and worthy of further study in patients with and without immediate reconstruction.
RADCOMP Trial[14]	Randomized open label (ongoing)	A pragmatic randomized clinical trial of patients with locally advanced breast cancer comparing proton and photon beam therapy.	Conventional fractionation (once a day, 5 days a week, for 5 to 7 weeks).	Measuring the effectiveness of proton therapy vs. photon therapy, including cardiovascular morbidity and mortality, health-related quality of life, cancer control outcomes, radiation dose and cardiac toxicity, disease control, and long-term survival at 5 and 10 years.
DBCG Proton Trial [15]	Randomized open label phase II (ongoing)	A randomized trial comparing standard photon RT to experimental proton RT.	Comparing proton radiation therapy and photon radiation therapy.	Radiation-associated ischemic and valvular heart disease at 10 years. Secondary outcomes include acute and late radiation morbidity, distant failure, secondary malignancy, and patient-reported outcomes.
PARABLE Trial [16]	Randomized open label phase III (ongoing)	Patients randomized to tailored photon radiotherapy or PBT, both receiving hypofractionated radiotherapy.	Total dose of 40 Gy in 15 fractions over 3 weeks.	Patient advocate feedback regarding the importance of considering the consequences of radiotherapy and patient-reported normal tissue toxicity in the breast measured 2 years after radiotherapy.

Safety and efficacy of proton therapy

Unlike traditional radiation techniques, proton therapy emerges as a promising and safer alternative to breast cancer radiotherapy, offering a beacon of hope for patients seeking effective treatment with minimal side effects. Studies consistently demonstrate its safety by showcasing a lower incidence of side effects such as skin irritation, fatigue, and damage to surrounding tissues. Proton therapy's targeted approach is particularly beneficial for left-sided breast cancers, where it significantly diminishes the risk of complications to critical organs like the heart. The resulting improvement in patients' quality of life and long-term outcomes underscores the safety advantages of proton therapy in breast cancer treatment [17].

A prospective proton therapy trial, enrolling 69 patients undergoing treatment of the breast or chest wall and regional lymph nodes, designated its primary endpoint as the incidence of severe radiation-induced complications, specifically grade ≥3 radiation pneumonitis or any grade 4 toxicity within a three-month post-radiotherapy window. Impressively, this investigation reported a complete absence of grade ≥3 pneumonitis events, thereby emphasizing the safety profile of proton therapy. Furthermore, protons exhibit notable proficiency in mitigating radiation exposure to the lungs, both concerning the low- and high-dose components. It is pertinent to note that dose modeling studies have proposed a diminished risk of second lung cancer with proton therapy relative to contemporary photon radiotherapy modalities [15].

Cardiac sparing and deep inspiration breath hold (DIBH)

Ongoing trials explore proton therapy's cardiac protection in breast cancer, refining protocols and assessing long-term outcomes. Advanced imaging and planning techniques offer valuable insights, highlighting proton therapy's potential to revolutionize breast cancer radiotherapy.

In the pursuit of optimizing cardiac sparing during breast cancer radiotherapy, the integration of DIBH has emerged as a valuable technique. DIBH involves instructing patients to take a deep breath and hold it during treatment, displacing the heart away from the radiation field. When coupled with proton therapy, DIBH enhances cardiac sparing by synchronizing the delivery of proton beams with the patient's breath-holding pattern. This dynamic combination ensures precise targeting of breast tumors while minimizing radiation exposure to the heart. The incorporation of DIBH in proton therapy for breast cancer underscores the commitment to patient safety and the continual refinement of treatment protocols to achieve the best possible outcomes [18].

**Figure 1:**
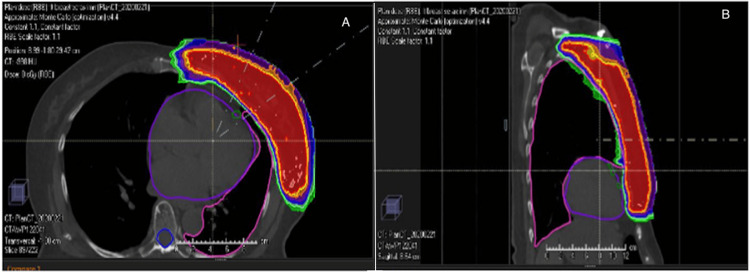
(A) Axial and (B) sagittal views of dose color wash from proton beam therapy plan showing very precise dose distribution to breast and regional nodal target areas while maximum sparing of the lung, heart, and contralateral breast [20]

Regarding the heart's physical displacement during DIBH, the use of low-density lung tissues minimizes alterations in the water-equivalent path length to the heart. Consequently, without significant inferior cardiac displacement, proton therapy may not yield substantial cardiac-sparing benefits. However, proton radiotherapy's en face proton fields maintain consistent proton path lengths despite respiratory motion [19]. The ongoing Danish Breast Cancer Group proton trial has evaluated that the proton planning strategy consistently produced robust treatment plans, meeting predefined criteria with a MHD of ≥4 Gy and/or lung V17Gy/V20Gy ≥37%, making 22% of patients eligible while considering target coverage and minimizing organ exposure (Figure 1) [16,20].

Dose distribution and critical structures

Adjuvant postmastectomy radiation therapy, targeting the chest wall and regional nodes, faces challenges in accurate dose determination due to complex geometry. Thin chest walls pose challenges where precise calculations are crucial. Advanced algorithms like Monte Carlo and Acuros XB align better with experimental data. Dose calculation parameters for patients with thin chest walls are sensitive to various factors, with partial bolus application improving outcomes for all algorithms. Patients with 20 to 25 mm of chest wall thickness exhibit sufficient target coverage without a bolus, reducing dependence on patient geometry and calculation algorithms. Accurate dose determination in these cases requires advanced algorithms and high resolution, with specific criteria or partial bolus implementation facilitating plan development and evaluation [21].

Postoperative radiation therapy reduces local relapse and breast cancer mortality but may increase late cardiovascular and secondary malignancy risks. Advanced techniques like conformal radiotherapy and IMRT lower cardiac and lung irradiation. PBT offers distinctive depth-dose distributions with a low to median entrance dose, a concentrated high-dose region (Bragg peak) in the tumor area, and a rapid decline to zero-dose distal to the target. This results in highly conformal and homogeneous physical dose distributions (Table 2). In a study comparing proton therapy with IMRT and conventional plans, mean doses to the ipsilateral lung and heart were lower with protons. Additionally, proton therapy substantially reduced the dose to the contralateral breast compared to IMRT [22].

PBT could be applied for simultaneous integrated boost delivery during whole breast irradiation, reducing the overall treatment time by 1.5 to 2 weeks. Higher fractional boost doses with proton therapy (approximately 2.2-2.4 CGE-Gy/fraction) lead to a higher BED to the target volume. As the dose distributions are highly conformal with both IMRT and protons, OARs not directly surrounding the target regions, such as the heart and lung, are not at greater risk for late toxicity with concomitant boost delivery. This approach enhances local control without increasing the risk to surrounding healthy tissues [22].

The mean lung dose (MLD) and volume receiving 5 Gy (lung V5), MHD and volume receiving 5 Gy (heart V5), maximum dose of the spinal cord (Dmax), and skin, as well as the mean and maximum dose of the esophagus, exhibited statistically significant improvements with PBT across all locations, surpassing the outcomes of all alternative photon plans in simulation (Tables 3-4) [23,24].

**Table 3:** Dosimetric advantages of proton therapy in breast cancer MHD: mean heart dose, MLD: mean lung dose, OAR: organs at risk [24]

OARs	Dose reduction	Potential benefits
Heart	10-fold reduction in cardiac and cardiac substructure dose exposure, 1.6 Gy lower MHD	Greater dosimetric advantage, comprehensive coverage of internal mammary nodes, advantageous in young patients and those with pre-existing cardiac risk factors
Lung	Absolute median differences in MLD ranged from 1.0 to 1.25 Gy	Very low instances of grade 2 radiation pneumonitis, no reported grade 3 pneumonitis, delayed risk of pulmonary fibrosis
Contralateral breast	No meaningful radiation exposure	Lower cancer risks in young women and those with challenging anatomy
Intrinsic muscles of the shoulder and chest	Lower doses to shoulder and chest muscles	Allows treatment without limiting therapeutic doses, prevents post-surgical complications

**Table 4:** Comparison of typical dose constraints for OARs in breast cancer radiation therapy Note: These dose constraints are commonly used targets in clinical practice and are based on established principles within the field of radiation oncology. Actual values may vary depending on institutional protocols and individual patient factors. OAR: organs at risk, MLD: mean lung dose

OAR	Proton therapy	Photon therapy
Heart	Mean dose <2 Gy	Mean dose <4 Gy
	V25 <10%	V25 <30%
Lungs	Mean dose <7.5 Gy	MLD <20 Gy
	V20 <15-20%	V20 <30%
Contralateral breast	Mean dose <0.005 Gy	Mean dose >2.1 Gy
	V5 <50%	V5 <50%
Skin	D1% = 108-109%	D1% ≥112%
Esophagus	Dmean 4.5 Gy	Dmean 7 Gy

Skin toxicity with proton beam therapy

The comparison between proton therapy and photon therapy (3D-CRT) for breast cancer patients reveals significant outcomes in the context of skin toxicity. A multi-institutional prospective study suggested that proton therapy resulted in a higher incidence of long-term skin toxicities (telangiectasia, pigmentation change, and others) for stage I breast cancer patients compared to 3D-CRT. However, the seven-year local failure rate did not significantly differ between the two cohorts [25].

A separate analysis from the National Cancer Database found no statistically significant difference in the five-year overall survival between proton and photon therapies for stage 0-III breast cancer patients. On the contrary, a single institutional study revealed that patients treated with PBS proton therapy had a higher incidence of acute grade ≥2 radiation dermatitis compared to photon therapy for stage IA-IIIC breast cancer, despite no observed statistically significant differences in skin dose [25].

Dosimetric comparison studies indicate that proton therapy delivers significantly higher skin doses for patients with an intact breast, while in the post-mastectomy setting, both protons and photons with a bolus show no significant skin sparing. Notably, in the supraclavicular field outside the bolus, photons exhibit significant sparing compared to protons. The long-term clinical impact on cosmetic outcomes remains to be determined, indicating the need for careful counseling for patients receiving proton therapy, particularly regarding higher skin doses in specific scenarios [26].

 Patient-centered care and breast reconstruction

Breast cancer affects one in eight women throughout their lives, but advancements in treatment, screening, and awareness have led to a consistent decrease in breast cancer-related mortality rates. The consideration of body image is crucial for women, influencing decisions regarding mastectomy. Reconstruction procedures are chosen to minimize the impact on body image [27].

A study examines factors influencing outcomes post-mastectomy and reconstruction. The inclusion of radiation after reconstruction is linked to an increased risk of a less favorable outcome, while avoiding radiation reduces this risk. Implant-based reconstructions with tissue expanders or direct-to-implant are associated with a higher risk of a poorer outcome. In contrast, most autologous reconstruction techniques, including the transverse rectus abdominis flap (TRAM), deep inferior epigastric perforator flap (DIEP), latissimus dorsi flap, and superficial inferior epigastric artery flap (SIEA), are linked to a lower risk. However, the gluteal artery perforator flap increases the risk, and mixed implant and autologous reconstruction procedures have a minor impact [27].

Satisfaction after reconstruction is negatively affected by radiation, implant-based reconstruction with tissue expanders or direct-to-implant, and a gluteal artery perforator flap. Conversely, satisfaction is improved with autologous reconstruction using DIEP, TRAM, or SIEA, no radiation, simple mastectomy, and bilateral reconstruction. Decisions regarding mastectomy and reconstruction in breast cancer treatment should factor in the influence on body image. Elements such as radiation and specific reconstruction techniques significantly contribute to determining outcomes and patient satisfaction. Autologous reconstruction methods yield better outcomes, whereas radiation and specific implant-based approaches are associated with increased risks of a less favorable outcome and decreased satisfaction [27].

**Figure 2:**
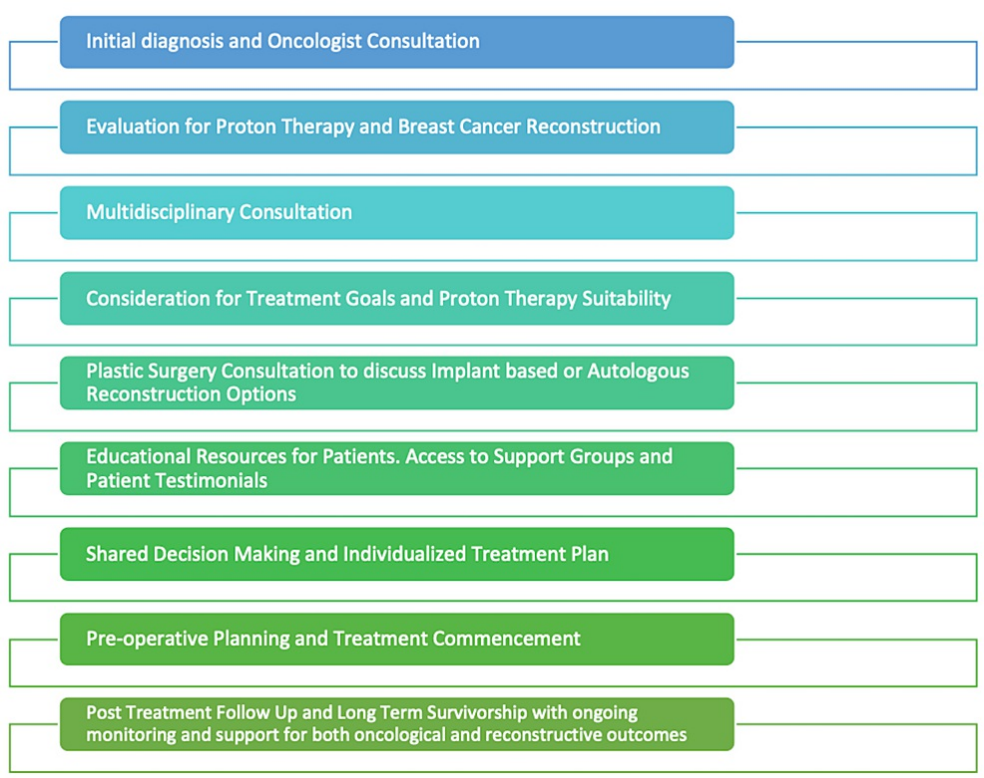
Patient decision-making flowchart: breast reconstruction in conjunction with proton therapy Image Credit: Hadia Fatima

PCC plays a significant role in the medical care of breast cancer patients, particularly in developing outcome measures. PCC emphasizes the importance of considering an individual patient's context in disease treatment (Figure 2). The rise of "evidence-based medicine" led to a growing interest in PCC, driven by dissatisfaction with impersonal approaches. In contrast to previous attempts to precisely define PCC, the current approach embraces the concept's heterogeneity, using it as an overarching term for healthcare that aims to recognize the individual within the patient. Efforts are now focused on justifying measures to implement PCC for breast cancer patients by demonstrating tangible, real-world effects [28].

A study involving 445 breast cancer patients found that those who underwent IBR reported significantly higher scores in psychosocial, sexual, and physical well-being compared to those without IBR. Multivariate regression analysis confirmed IBR as an independent factor for better patient-reported quality of life. The findings support recommending IBR discussions for all mastectomy candidates to facilitate shared decision-making [29].

A study by Ho et al. investigated factors influencing satisfaction in breast reconstruction patients [29]. The mean age was 54.3±9.3 years, and the mean body mass index was 25.2±4.3. Multivariate analysis revealed that satisfaction with preoperative information and the plastic surgeon significantly predicted higher satisfaction with breasts and overall outcomes. The findings underscore the importance of PCC, suggesting that improving information delivery and the surgeon-patient relationship could optimize outcomes in breast reconstruction patients [30].

The ethical analysis examines the appropriateness of combining IBR with post-mastectomy radiotherapy (PMRT), a practice linked to increased complications. Evaluating principles like beneficence, non-maleficence, autonomy, and justice, the study suggests that choosing IBR or PMRT, delayed autologous reconstruction, or no reconstruction are ethically valid options. However, considering the benefits and harms, IBR may pose ethical concerns, as alternatives could offer similar benefits with fewer complications. Denying IBR doesn't undermine autonomy, but biases may influence patient decisions. IBR with PMRT may raise justice concerns by potentially diverting care from other patients, and cost-effectiveness considerations should be conservative. In conclusion, ethical considerations suggest caution in offering IBR with expected PMRT based on current knowledge and established healthcare norms (Figure 2) [31].

Breast reconstruction techniques

In the planning of reconstructive procedures after mastectomy, the timing of either immediate or delayed repair is a crucial consideration. Immediate repair, performed during the same surgery as cancer resection, offers the advantage of utilizing a more natural skin envelope, especially in skin/nipple-sparing mastectomy. However, oncologic factors such as cancer stage and BRCA mutation status, as well as the need for adjuvant therapy like radiation, influence the feasibility of immediate repair. Patients requiring radiation are often better suited for autologous tissue reconstruction to minimize complications. On the other hand, delayed reconstruction involves a separate surgery for reconstruction, giving patients time to decide whether to undergo reconstruction or undergo adjuvant radiation. The use of alloplastic implants for breast restoration is explored further in various studies, providing options for both immediate and delayed situations [32].

IBR offers advantages such as fewer surgeries, improved cosmetic outcomes, and the avoidance of the emotional impact of breast loss. However, drawbacks include the risk of postoperative skin envelope necrosis and the potential for the reconstructed breast to harden with postoperative radiation therapy. In contrast, delayed reconstruction allows more time for decision-making on reconstruction methods and avoids additional cancer therapies. Downsides include an increased number of surgeries and potential skin hardening after radiation therapy (Table 5). The evolution of breast reconstruction, marked by developments like silicone gel implants, autologous tissue transfer, tissue expanders, perforator flaps, and fat grafting, has made the procedure less invasive and more versatile over the decades [33].

**Table 5:** Comparative aspects of breast reconstruction options [37]

Type of reconstruction	Recovery period	Potential complications	Other considerations
Implants	Shorter	Infection, seroma, hematoma, blood clots, extrusion, implant rupture, scar tissue	Not suitable after chest radiation, inadequate for large breasts, not lifelong, increased risks (obesity, diabetes, smoking)
Autologous tissue	Longer (initial)	Tissue necrosis, increased blood clot risk, pain, and weakness at the donor site	More natural breast shape, softer feel, replaces damaged tissue, leaves donor site scar (pedicled flap shorter, free flap longer and more technical)

The common approach to breast reconstruction involves placing implants beneath the chest muscles, causing potential issues like pain, reduced shoulder motion, and chest wall deformities. However, the pre-pectoral procedure, considered the most minimally invasive option, allows for mastectomy through a small incision under the breast. A tissue expander is placed on top of the muscle, creating a breast mound, and can later be replaced with an implant through the same incision. This technique aims to mitigate complications associated with traditional reconstruction methods [34].

The CHARM Trial, a phase II ongoing study, examines outcomes in hypofractionated radiotherapy with two-stage expander/implant reconstruction. It focuses on reconstruction failure, cosmetic outcomes, and radiotherapy side effects at 12 months post-reconstructive surgery [35]. The FABREC Trial, an ongoing randomized open-label study, compares hypofractionation to conventional radiation therapy in post-mastectomy IBR, evaluating outcomes at six and 18 months and oncologic outcomes at 10 years [36]. The findings from these ongoing trials will contribute valuable insights for future dedicated research on the use of hypofractionated proton therapy in the treatment of breast cancer.

Innovative planning strategies and challenges with tissue expanders

Tissue expanders are indispensable in plastic and reconstructive surgery, particularly in breast reconstruction following mastectomy or corrective procedures for congenital anomalies. Their primary role is to create space and stretch surrounding tissues in preparation for the eventual placement of a permanent implant, contributing significantly to the restoration of a natural and aesthetically pleasing breast shape. These medical devices are typically constructed from biocompatible materials, with silicone and saline being the main types used [38].

Like silicone breast implants, silicone tissue expanders are filled with a silicone gel known for its durability and flexibility. This design facilitates the controlled expansion of surrounding tissues, offering a smooth and natural feel that patients often prefer. On the other hand, saline tissue expanders are filled with sterile saline solution and encased in a silicone shell. This design allows for adjustability in the volume of saline, a feature beneficial during the expansion process [39].

Placement options for tissue expanders include submuscular placement under the pectoralis muscle, providing a protective barrier for healing skin, or prepectoral placement over the muscle with an acellular dermal matrix offering additional support during skin recovery. The mesh gradually absorbs into the body without requiring removal. Over weeks to months, the expander is filled with either saline or silicone gel through a valve or port, gradually stretching the skin and underlying tissues to create a pocket for the permanent breast implant. Tissue expanders, through controlled and gradual expansion, minimize trauma and offer customization based on patient preferences, making them versatile tools for effective preparation in various reconstructive procedures [40].

While generally well-tolerated, patients considering breast reconstruction with tissue expanders should engage in discussions with their plastic surgeons to determine the most suitable choice based on individual factors such as preferences, medical history, and the surgeon's expertise.

In a substantial prospective study conducted by Cordeiro et al., involving 1415 patients undergoing two-stage implant-based breast reconstruction with postmastectomy radiation therapy to the permanent implant, the findings revealed a significant impact of radiation on reconstruction outcomes. With a mean follow-up of 56.8 months, the study reported a notable 9.1% implant loss rate in irradiated implants compared to 0.5% in nonirradiated ones. Despite this challenge, the majority of irradiated patients maintained good to excellent aesthetic results, with 92% expressing satisfaction. This underscores the resilience and positive long-term outcomes of immediate tissue expander/implant reconstruction, even in the context of postmastectomy radiation therapy [41].

Tissue expanders are integral to two-stage IBR but introduce challenges in treatment planning due to metallic ports causing artifacts on CT images. Innovative planning strategies, including metal artifact reduction algorithms and Monte Carlo methods, have enhanced dose calculation precision in proton therapy planning, resulting in superior dosimetric characteristics compared to photon-based plans.

In a phase II clinical trial involving proton therapy for patients undergoing mastectomy with immediate implant-based reconstruction, outcomes demonstrated notable merit. Twenty-eight percent of patients experienced radiation therapy-related complications, while a mere 4% encountered reconstructive loss, presenting a favorable contrast to recent reports of photon therapy outcomes from the same institution [10].

Current data further indicates a significant difference in tissue expander loss between patients receiving radiation to the tissue expander and those receiving radiation to the final implant (8.5% vs. 1.0%). The extended duration of tissue expander placement in the tissue expander-external beam radiotherapy group (13.2 months) versus the implant-external beam radiotherapy group (6.2 months) may contribute to this disparity. Delaying the exchange procedure for six months aims to reduce complications during the acute radiation phase. Univariate analysis suggests a higher tissue expander-external beam radiotherapy reconstructive failure rate (18.1% vs. 12.4%), and multivariable analysis indicates higher odds of failure with radiation to either the tissue expander or the implant compared to non-external beam radiotherapy patients. Those receiving radiation to the tissue expander face a 10% higher likelihood of overall reconstruction loss than the implant-external beam radiotherapy group. Consistent with other studies, Kaplan-Meier analysis projects a significantly higher potential for TE-external beam radiotherapy failure (32.0% at six years vs. 16.4% in implant-external beam radiotherapy), supporting the recommendation to radiate the final implant to minimize reconstructive failure [42].

Multidisciplinary management of breast cancer

Breast cancer treatment, a multimodal approach combining surgery, radiotherapy, and chemotherapy, aims to reduce mortality rates but has side effects. Multidisciplinary management optimizes therapeutic effectiveness while minimizing toxic effects, with medical, surgical, and radiation oncologists collaborating on personalized treatment plans. Oncoplastic surgery enhances oncological and cosmetic outcomes but complicates decisions on PMRT and reconstruction timing. Radiation increases complications in implant reconstructions, necessitating patient education on therapy implications. Challenges in radiation planning for oncoplastic surgery highlight the need for careful consideration. Intraoperative radiation therapy and neoadjuvant radiation therapy offer strategies in specific cases. The multidisciplinary approach, with patient involvement, balances treatment effectiveness and cosmetic outcomes, promising improved patient care and outcomes [43,44].

Integrating hypofractionated proton therapy with breast reconstruction

The integration of hypofractionated proton therapy with breast reconstruction signifies a significant leap forward in comprehensive breast cancer care. This integration enables clinicians to not only effectively eliminate tumors but also preserve aesthetic outcomes and enhance patient quality of life. Hypofractionated proton therapy delivers precise radiation, minimizing harm to healthy tissues and reducing long-term complications like radiation-induced fibrosis [45]. When combined with breast reconstruction, proton therapy minimizes radiation-related changes to breast tissue and implants, thus improving patient satisfaction and psychological well-being [46]. Additionally, the potential decrease in radiation-induced toxicities facilitates a smoother recovery for breast cancer survivors undergoing reconstruction [47]. However, integrating these modalities poses logistical challenges in coordinating treatment schedules and considering the impact on tissue healing and financial burdens [48]. Addressing these challenges necessitates close collaboration between radiation oncologists and reconstructive surgeons, emphasizing the importance of multidisciplinary care and ongoing research to refine treatment protocols and optimize outcomes for breast cancer survivors [49]. Ongoing research in hypofractionated proton therapy for breast cancer is focused on refining treatment protocols, investigating optimal patient selection criteria, and exploring combination therapies. Clinical trials are underway to evaluate the efficacy of hypofractionation in different breast cancer subtypes and patient populations, aiming to establish proton therapy as a standard of care in breast cancer treatment.

## Conclusions

Hypofractionated proton therapy for breast cancer offers promising outcomes despite the potential for side effects such as skin irritation, fatigue, and changes in breast texture, which are generally less frequent than those associated with traditional radiation therapy. This treatment modality demonstrates efficacy and shorter treatment durations, with studies suggesting favorable cosmetic results and reduced complications in breast reconstruction compared to conventional radiation therapy. Nevertheless, further research is needed to fully evaluate its long-term impact on breast reconstruction outcomes.

The convergence of hypofractionated proton therapy and breast reconstruction represents a significant advancement in breast cancer patient care. Achieving a careful equilibrium between innovation and patient-centric decision-making is crucial to providing the best treatment strategies and results. The collaboration between these fields holds promise for mitigating radiation-induced complications, particularly in cardiac and pulmonary preservation. Ongoing investigations into combining proton therapy with techniques like DIBH for cardiac protection signify a hopeful future for personalized and precise breast cancer treatment. By embracing these advancements, we can ensure an improved quality of life and better outcomes for patients.
